# Multi-scale sensorless adaptive optics: application to stimulated emission depletion microscopy

**DOI:** 10.1364/OE.393363

**Published:** 2020-05-19

**Authors:** Jacopo Antonello, Aurélien Barbotin, Ee Zhuan Chong, Jens Rittscher, Martin J. Booth

**Affiliations:** 1 Department of Engineering Science, University of Oxford, Oxford OX1 3PJ, UK; 2 Institute of Biomedical Engineering, Old Road Campus Research Building, Oxford OX3 7DQ, UK

## Abstract

Sensorless adaptive optics is commonly used to compensate specimen-induced aberrations in high-resolution fluorescence microscopy, but requires a bespoke approach to detect aberrations in different microscopy techniques, which hinders its widespread adoption. To overcome this limitation, we propose using wavelet analysis to quantify the loss of resolution due to the aberrations in microscope images. By examining the variations of the wavelet coefficients at different scales, we are able to establish a multi-valued image quality metric that can be successfully deployed in different microscopy techniques. To corroborate our arguments, we provide experimental verification of our method by performing aberration correction experiments in both confocal and STED microscopy using three different specimens.

## 1. Introduction

Fluorescence microscopy enables targeted, high-resolution imaging in specimens and its use is fundamental for the advancement of biological sciences [1]. Nevertheless, the scope of this technique is limited in some scenarios, especially when applied to image deep into optically heterogeneous media such as tissue. In this case, the presence of aberrations detrimentally affects the contrast and quality of the data that can be recorded [2]. This situation is further exacerbated when employing super-resolution microscopes [3], where overcoming the diffraction limit is contingent upon accurate shaping of the microscope point-spread functions (PSF), see for example [4–6]. To overcome this impediment, one can implement adaptive optics [2] (AO), whereby wavefront modulation is applied in order to minimise aberrations.

A critical issue in successfully deploying AO is the problem of aberration measurement. Direct aberration measurement schemes can be adapted to a particular microscopy technique on a case by case basis, but require significant alterations to the original design of the microscope. For example, in [7], a lattice light sheet microscope is augmented with additional optical trains and a laser source, which are dedicated solely to AO, leading to inflation in the cost and complexity of the overall system.

A less invasive approach entails implementing indirect aberration measurement methods [2], where the aberration is deduced by examining the images obtained from the microscope itself, instead of relying on a dedicated sensor. In practice, indirect measurement can be achieved by iteratively maximising a metric that quantifies the quality of an image. Previous research has focused on identifying which metrics are most effective for a number of different microscopes, see [2] and the references therein. As it turns out, a successful metric for one microscopy technique may be sub-optimal for others. For instance, maximising the image brightness is an effective metric for two-photon and confocal microscopy [8,9], but may undermine the resolution in stimulated emission depletion (STED) microscopy [3]. This is motivated by the fact that efficient depletion in STED is accompanied by a reduction in the brightness of images [4,10]. On the other hand, it is clear that the presence of aberrations adversely impacts the resolution regardless of the particular microscope at hand [11] and, as a result, it seems reasonable to expect that a universal image quality metric could be identified – one that is equally effective for all types of microscopes.

This paper represents an advancement towards the ambitious goal outlined above. Here we propose a novel method to measure aberrations that is based on multi-scale wavelet analysis [12]. Wavelets have been employed in the past to solve denoising problems in microscopy [13,14] and in sensor-based adaptive optics systems [15]. Nonetheless, to the best of our knowledge, the use of wavelets to measure aberrations in fluorescence microscopy has not been previously reported. Contrary to conventional Fourier analysis, wavelets are able to detect spatially localised variations in the frequency content of images. As such they represent an ideal tool to quantify the effect of aberrations on the resolution, regardless of the particular microscopy technique.

The paper is organised as follows. In Section 2, we briefly introduce some key concepts from wavelet analysis. We outline how this latter can be used for our purposes in Section 3 and Section 4. We describe our custom-built STED microscope in Section 5 and report our experimental results in Section 6.

## 2. Comparison of Fourier and wavelet analysis

To the best of our knowledge, Fourier analysis has been at the foundation of almost every attempt to quantify image quality in the current literature relevant for AO in microscopy. For example, the total image brightness, i.e., the zero frequency component, has proven to be an effective metric to correct aberrations in confocal [8] and two-photon microscopy [9]. Similarly, maximising the low spatial frequency content of images was shown to be ideal for some incoherent imaging systems [16]. Finally, a metric combining brightness and sharpness [17] and a metric based on Fourier ring correlation [18] (FRC) were found to be suitable for STED microscopy [10,17,19].

**Fig. 1.**
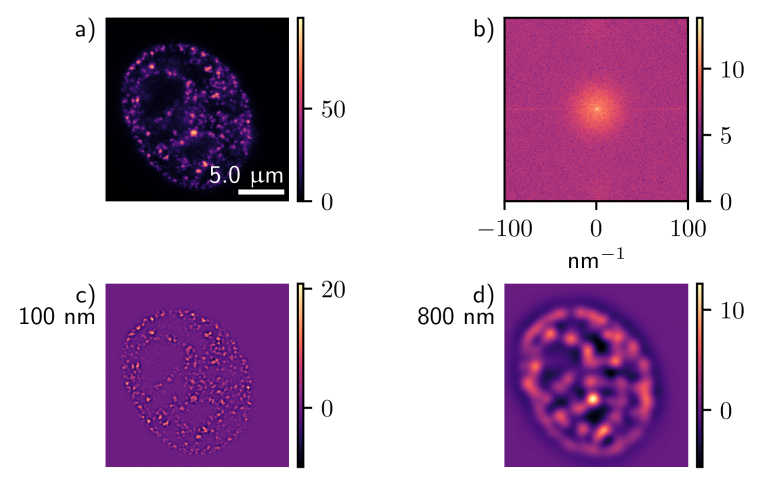
Comparison of the Fourier and wavelet analyses of an image. (a) 2D STED image (raw photon counts; 400×400 pixels; HeLa cell nucleus); (b) magnitude of the Fourier spectrum of the image in logarithmic scale. (c) and (d) wavelet coefficients at scales of 100 and 800 nm, respectively. Linear scale.

A primary concern about using Fourier analysis for our purposes is that it is not an ideal tool to detect spatially localised variations in the frequency content of images, a fact that stems directly from Heisenberg uncertainty [12]. In fluorescence microscopy, images typically consist of sparsely labelled structures of interest that are embedded in an ideally uniform background – for example, consider an image of a HeLa cell nucleus obtained with our STED microscope, which is shown in Fig. 1(a). Here the informative component of the image is primarily located in the labelled structures, whereas noise contributions are dominant elsewhere. The Fourier analysis of this image is reported in Fig. 1(b), in logarithmic scale. Each coefficient of the Fourier analysis is associated to a complex exponential in the spatial domain that is not spatially localised, i.e., the complex exponential is non-zero both over the structures of interest and over the background. As a result, the informative content of the image is spread over a large portion of the Fourier spectrum, and contributions due to the informative part of the data cannot be easily discerned from contributions due to noise.

Using wavelets, instead, one is able to restrict the frequency analysis to locations of interest within an image [12]. In fact, wavelets allow for an analysis that is simultaneously localised both in the spatial and in the frequency domain, albeit within the lower bound of minimal Heisenberg uncertainty [12]. This is illustrated by Fig. 1(c), which shows the wavelet coefficients obtained for a fixed wavelet scale of 100 nm. The choice of the wavelet scale determines which particular set of frequencies is sampled from the original image of the cell [12]. At the same time, spatial information is also retrieved, as each pixel within Fig. 1(c) corresponds to a particular location within the spatial domain. A different sampling in the frequency domain is obtained by changing the scale of the wavelet. For instance, Fig. 1(d) shows the coefficients obtained for a scale of 800 nm.

The simultaneous spatial and frequency localisation capability of wavelets render them an ideal tool to detect changes in the image resolution that are due to aberrations. As finer features in an image become indiscernible due to increasing aberrations, one can observe a corresponding decreasing magnitude for wavelet coefficients at finer scales and increasing magnitude for the ones at coarser scales.

## 3. Definition of a multi-scale image quality metric

In this section we define a multi-scale image quality metric that is based on wavelet analysis. Among many possible choices for the wavelet function present in the literature [12], we opted for the starlet wavelet, which is defined in [20,21]. The motivation for our choice is two-fold. First, the starlet wavelet has been previously employed in denoising applications for images affected by low photon count Poisson noise [22], which is relevant for fluorescence microscopy. Second, the starlet wavelet is well-suited for the analysis of mostly isotropic objects, as commonly found in astronomy and biology applications [20]. Nonetheless, we remark that one can trivially adapt the procedure presented below to the case when other wavelet functions are selected.

Given an image I of dimensions N×N, one can compute the starlet analysis [20,21] by repeated application of the following equations,

(1) $$\begin{array}{ll} a_{j} & =h^{↑j-1}*a_{j-1},\\ d_{j} & =a_{j-1}-a_{j}, \end{array}$$

where $a_{j}$ and $d_{j}$ are the approximation and detail coefficients at scale j, and we let $a_{0}:=I$. Each wavelet coefficient consists of an N×N matrix, where the position of an element within the matrix identifies a particular location in the spatial domain, whereas index j identifies the scale of the wavelet, which is related to its frequency content as outlined earlier. Note that throughout the paper we refer to both the matrices and the elements within them interchangeably as wavelet coefficients. Above, ∗ denotes the convolution operation, whereas $h^{↑j-1}$ is a 2D discrete filter that results from applying the *à trous* algorithm as outlined in detail within [21].

From the set of detail coefficients $d_{j}$, we first define a vector m′ that collects the squared magnitude of each coefficient, i.e.,

(2) $$m\prime :=\left[\begin{array}{c} \sum_{k}{\left|{\left(d_{1}\right)}_{k}\right|}^{2}\\ \vdots \\ \sum_{k}{\left|{\left(d_{J}\right)}_{k}\right|}^{2} \end{array}\right],$$

where $\sum_{k}{\left(d_{j}\right)}_{k}$ denotes the sum of the $N^{2}$ elements ${\left(d_{j}\right)}_{k}$ of $d_{j}$, which are enumerated using a single index k for simplicity. The size of m′ is J, which is determined by the maximum level selected for the wavelet decomposition in Eq. (1). Next we define the normalised vector

(3) m:=m′/|m′|,

which can be regarded as a vector-valued image quality metric. As the magnitude of the aberrations increases, the resolution of I deteriorates. This can be detected as a change in the relative ratios among the elements of m, which are subject to the unit normalisation constraint. An increase in the magnitude of the aberrations leads to smaller elements of m corresponding to finer scales. At the same time, the magnitude of the elements of m corresponding to coarser scales increases. Note that vector m, as well as the wavelet coefficients $a_{j}$ and $d_{j}$, are in fact meant as functions of the residual aberration in the system. In order to keep a simple notation, we do not make this dependency explicit throughout the paper.

**Fig. 2.**
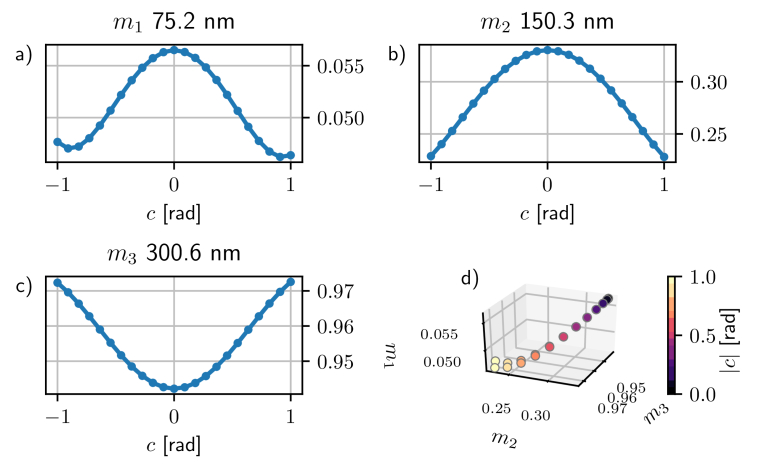
Simulation results showing the sensitivity of vector m to the residual aberration c. For each value of c, an aberrated image is generated (see Section 3) and the corresponding value of m is computed. (a)–(c) elements of m corresponding to scales 75.2, 150.3, and 300.6 nm; (d) evolution of m as c varies between −1 and 1. The scalebar on the right indicates the residual rms, i.e., |c|. As |c| increases, the finer scales elements $m_{1}$ and $m_{2}$ decrease whereas the coarse scale element $m_{3}$ increases.

The sensitivity of m to the residual aberration is illustrated with a simulation in Fig. 2, where an incoherent imaging system is modelled using scalar Fourier optics [11]. We consider part of the specimen shown in Fig. 1(a) as an object that is emitting fluorescence at 690 nm and is imaged via a 1.4 NA lens, similarly to the imaging parameters of our experimental setup in Section 5. The pixel size of the image is set to a quarter of the Airy disk radius. A random combination of Zernike aberrations [23] with Noll indices between 5 and 11 is chosen by drawing a unit vector r, and scaling it by a scalar c, which varies between −1 and 1. The first three detail coefficients $d_{1}$, $d_{2}$, and $d_{3}$, are then computed for each of the aberrated images, along with the corresponding vectors m. Note that, for illustration purposes, we restrict the wavelet analysis such that it results in a three-element vector m. In the more general case, instead, the maximum depth of the wavelet analysis is limited by the base two logarithm of the smaller dimension of the image in pixels. Figures 2(a)–(c) reports the variation of the elements of m as a function of the residual aberration c. It can be seen that as the aberration root-mean-square (rms) increases in magnitude, a drop is recorded for elements $m_{1}$ and $m_{2}$, which correspond to the finer wavelet scales of 75 and 150 nm. This is accompanied by a simultaneous increase of element $m_{3}$, corresponding to the coarsest scale considered in the wavelet analysis. The same phenomenon can also be observed by looking at the three-dimensional plot in Fig. 2(d), which shows the evolution of the tip of vector m as a function of the parameter c. For larger residual rms |c|, vector m rotates away from the axes of the finer coefficients $m_{1}$ and $m_{2}$, indicating inferior resolution. The aberration correction problem can therefore be expressed as that of finding the optimal orientation of vector m, i.e., the one where the contributions due to wavelet coefficients with finer scales is maximised.

## 4. Aberration correction by multi-scale optimisation

At first sight, correcting aberrations by optimising the orientation of vector m may seem an intricate problem. First, the possible orientations that vector m can assume depend upon the fluorescent structures being imaged and the signal to noise ratio. For instance, consider the case where the structures exhibit features commensurable to the smallest scale that can be resolved with the microscope. Then the elements of m that correspond to the finest scales of the wavelet decomposition, say $m_{1}$ in Fig. 2, will be significant indicators for the residual aberration. Henceforth, maximising such elements may seem a sound strategy for aberration correction. This strategy, however, may be misguided when imaging coarser structures or in the presence of large aberrations, when one cannot reliably detect variations at the finest scales. Complications may also arise for low photon count scenarios, where the finest scales may become sensitive to Poisson noise. To avoid these pitfalls, one may consider maximising the elements of m at intermediate and coarse scales.

Instead of tackling the complex problem of optimising the orientation of m, we opt for a more practical heuristic approach. We propose a straightforward generalisation of the algorithm defined in detail in [24], which we denote as the PN algorithm. This latter is commonly employed to apply aberration correction by maximising a scalar-valued image quality metric. The PN algorithm applies aberration correction in an incremental fashion, i.e., by correcting a single aberration mode at a time. During correction of a mode, a series of P distinct correction amounts are applied and the corresponding images are recorded. At the same time, the values of the image quality metric are computed for each image. Finally, the optimal correction is found by fitting the computed values using a unimodal function and by finding its extremum. These steps are repeated for the subsequent mode until all the N aberration modes are corrected.

**Fig. 3.**
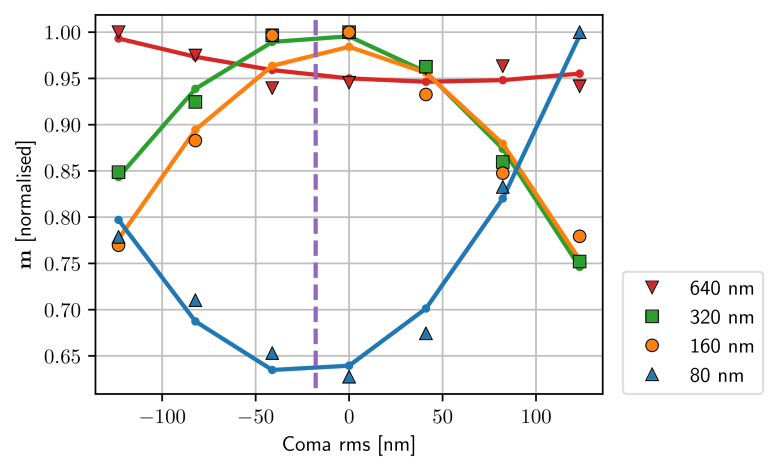
Fits of the elements of m obtained from one iteration of the generalised PN algorithm applied to correct coma (Noll index 7). The markers denote experimental data and the lines show the fits. The corresponding wavelet scales are reported on the right. The optimal correction to apply is computed as a linear combination of the extrema of each fit (see Section 4), and is indicated with a dashed line. This correction was obtained in our experiments in Section 6, see the STED correction in Fig. 7.

The PN algorithm can be generalised to our case by treating each element of m as an independent scalar-valued metric and by combining the optimal corrections computed for each element. Consider correcting a single aberration mode such as coma when P=7 and J=4. By following the original PN algorithm [24] we can obtain four fits, one for each element of m, as shown in Fig. 3. This leads to four possible aberration corrections, $c_{1},\ldots ,c_{4}$, obtained by finding the extremum of each fit. Sensible criteria to combine $c_{1},\ldots ,c_{4}$ into a single correction include, among other options, looking at the residuals and the concavity of each fit. In the experiments below we used an empirical formula to assign a score $s={\left(m_{\text{max}}-m_{\text{min}}\right)}^{3}/(m_{\text{max}}\epsilon )$ to each fit, where $m_{\text{max}}$ and $m_{\text{min}}$ are the maximum and minimum of the data points to fit and ϵ is the squared error of the fit. The single correction to apply was finally obtained as a weighted sum of the scores, i.e., $c=\sum_{1}^{4}s_{i}c_{i}/\sum_{1}^{4}s_{i}$. We remark that this procedure still entails taking P images per aberration mode, so that the total number of necessary images remains PN, as required by the regular PN algorithm [24].

Note that the element corresponding to the finest scale $m_{1}$ has a positive concavity in Fig. 3. This suggests that this element should be minimised to apply aberration correction, which is in apparent contradiction to what outlined above – compare with Fig. 2. In this case, $m_{1}$ happens to be more sensitive to Poisson noise rather than to fine features in the image, which is consistent with the conventional use of wavelets for denoising applications [22]. This possibility does not represent an issue for the algorithm outlined above, since the extrema of each fit are sought regardless of the sign of the concavity.

It is worth mentioning that, when using the FRC metric as in [19], one requires double the amount of measurements in total, i.e., 2PN. This is motivated by the fact that two images with independent noise realisations are necessary to compute a single evaluation of the FRC metric, which incurs in additional bleaching. Alternatively one must resort to more complex schemes whereby the two measurements are interleaved in space or time [25,26].

## 5. Description of the custom-built STED microscope

We validated the aberration correction method proposed in this paper using a custom-built STED microscope. This is an ideal system to showcase our proposed algorithm since one can adjust the resolution of the instrument by either operating it in confocal mode or in STED mode at different power levels. Our results demonstrate successful aberration correction using the same metric and parameters in both imaging modalities.

**Fig. 4.**
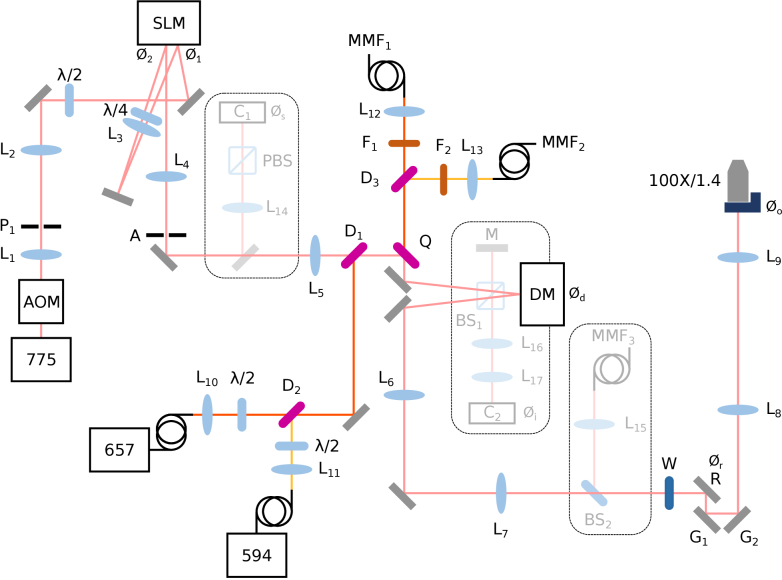
Layout of the adaptive STED microscope described in detail in Section 5. Legend: L lens; P pinhole; λ/2 half-wave plate; λ/4 quarter-wave plate; A aperture; C CMOS camera; PBS polarising beam splitter; D dichroic beam splitter; Q quad-band beam splitter; F emission filter; BS beam splitter; MMF multi-mode fibre; R resonant mirror; G galvanometer mirror; M flat mirror; W achromatic quarter-wave plate; ∅ conjugate pupil planes.

The layout of our setup is depicted in Fig. 4. Light from a 775 nm pulsed fibre laser (775; MPB Communications; PFL-80-3000-775-B1R; 80 MHz, 950 ps, 3 W) was incident onto an acousto-optic modulator (AOM) (AA Optoelectronics; MT110-B50A1.5-IR-Hk) and diffracted onto a spatial filter comprising an aspheric lens (L1; Thorlabs; AC080-020-B-MLP1) and a pinhole (P1; Thorlabs; P50CH). The beam was subsequently collimated by L2 (100 mm) and directed through a half-wave plate (λ/2), such that it eventually impinged onto a spatial light modulator (SLM) (Hamamatsu; X10468-02). A second reflection off the SLM was achieved by first directing the beam forwards through a quarter-wave plate (λ/4) and lens L3 (200 mm), and then backwards through the same elements after reflection off a flat mirror. This design, conceived in [27], enables 3D STED by allowing the simultaneous application of both the 2D (vortex) and Z (top-hat) phase masks using orthogonal polarisations states. Light diffracted at the second pass over the SLM was subsequently filtered by an aperture A to select the first order. The beam was then directed through a dichroic beam splitter (D1; Semrock; FF757-Di01), reflected off a custom quad-band beam splitter (Q; Chroma; ZT485/595/640/775rpc) [28], and impinged onto a deformable mirror (DM) (Boston Micromachines Corporation; Multi-3.5). The SLM and DM were conjugated by lens L4 and L5 (both 200 mm). A second conjugation between the DM and the scanners was achieved via lens L6 (300 mm) and lens L7 (250 mm). The scanners comprised an 8 kHz resonant mirror (R; Cambridge Technology; 8 kHz CRS) for the fast axis and two galvanometer scanners (G1 & G2; Cambridge Technology; 6220H). These latter moved synchronously acting as the slow axis [28]. The resonant scanner was conjugated to the back aperture (BA) of a microscope objective (100X/1.4; Olympus; UPlanSApo 100X NA 1.4 oil immersion) via lens L8 (150 mm) and L9 (250 mm). The objective was supported by an objective scanner (Physik Instrumente; P-725-4CD). An achromatic quarter-wave plate W was used to convert the polarisation states of the depletion to left and right circular polarisation.

Excitation was provided by two pulsed laser diodes, 657 (PicoQuant; LDH-P-C-650; 80 MHz, > 80 ps, 6.2 mW) and 594 (PicoQuant; LDH-D-TA-595; 80 MHz, > 92 ps, 6.2 mW), which were collimated by lenses L10 and L11 (both 60 mm), respectively. These beams were combined via a dichroic beam splitter (D2; Semrock; FF635-Di01), and merged into the main beam path via dichroic D1. Fluorescence emission was collected via the same objective and extracted from the main beam path via beam splitter Q. Fluorescence emission was subsequently divided into two channels by dichroic beam splitter D3 (Semrock; FF652-Di01). Channel one consisted of filter F1 (Chroma; ET690/50m), lens L12 (200 mm), and a 100 μm multi-mode fibre (MMF1). The second channel comprised a 615/30 nm filter (F2), lens L13 (200 mm) and a 100 μm multi-mode fibre (MMF2).

The system included additional branches that could be enabled for troubleshooting purposes and are highlighted within boxes in Fig. 4. A branch to examine the two passes over the SLM consisted of a flip mirror, lens L14 (300 mm), a polarising beam splitter PBS and a CMOS camera (C1; Thorlabs; DCC1545M). An interferometer for calibration of the DM could be activated and consisted of a flat mirror M, a 10R/90T beam splitter BS1 and a camera (C2; Thorlabs; DCC1545M). The DM and camera were conjugated by lenses L16 (100 mm) and L17 (60 mm). A branch to image back scattered light from the sample was implemented using a flip-in pellicle beam splitter (BS2), lens L15 (200 mm), and an 125 μm multi-mode fibre (MMF3).

The multi-mode fibres were connected to different channels of a single photon counting module array (Excelitas Technologies Corp.; SPCM-AQ4C). Synchronisation of the laser pulses and gated detection were obtained using two programmable delay boxes (Opsero; custom board). The trigger output of the 775 nm laser was converted to TTL (Pulse Research Lab; PRL-350TTL-NIM) and distributed (Pulse Research Lab; PRL-414B) to each box, along with the corresponding TTL output coming from the single photon counting module. Each box generated a delayed trigger for the excitation laser and the gated TTL signal corresponding to the photons detected by the avalanche photodiode (APD).

The microscope was operated via a desktop computer using custom software. A field-programmable gate array (FPGA) (National Instruments; PCIe-7852R) controlled the scanners, objective piezo, and AOM. The programme executing on the FPGA was compiled using LabVIEW and performed forwards line scans, during which it enabled the STED line at a selected power via the AOM and counted the photons detected by the APDs. At the same time, the programme coordinated movement of the galvanometer mirrors and objective piezo following a trigger signal generated by the resonant mirror driver box. The software running on the host computer comprised a front-end GUI written in Python, which also controlled the DM and SLM, and a back-end written in C. This latter streamed the waveforms for scanning to the FPGA and simultaneously retrieved the photon counts.

### 5.1 Optimisation of the double pass over the SLM

We followed the procedure in [29] to align the SLM in the depletion branch. Note that, since the beam is reflected twice over the SLM at $\emptyset_{1}$ and $\emptyset_{2}$, it accumulates static aberration due to both of these distinct locations over the SLM window. Nevertheless, phase modulation only occurs at one of these reflections for each polarisation state and, as a result, the manufacturer provided flattening for the SLM window is not applicable. See also [30]. Correct removal of all static aberration at $\emptyset_{1}$ and $\emptyset_{2}$ was instead achieved by maximising the intensity measured with a camera temporarily installed behind aperture A.

### 5.2 Calibration of the DM

The DM was calibrated with the interferometer branch in Fig. 4, which was enabled by inserting BS1. After tilting flat mirror M in the reference arm, one could extract the wrapped phase from a single interferogram using Fourier-based fringe analysis [31,32], and subsequently apply a phase unwrapping algorithm [33]. We obtained the influence matrix H of the DM as outlined in [34]. In our case the back aperture of the objective was imaged onto a smaller portion of the active area of the DM, which implied that some actuators at the boundary of the area had negligible phase contributions over the effective pupil of the microscope. For this reason we applied regularisation by defining a diagonal matrix $\Pi :=\lambda \mathrm{diag}(\beta_{1},\ldots ,\beta_{N_{a}})$ that penalises actuators outside the effective aperture. Here λ is a regularisation parameter which we set to $5\;\times 10^{-3}$ and $N_{a}$ is the number of actuators of the DM. We set the coefficients $\beta_{1},\ldots ,\beta_{N_{a}}$ to a value between zero and one, depending on whether the corresponding actuator lied within or outside the effective aperture. The control matrix C was finally computed by solving a regularised least-squares problem, i.e.,

(4) $$C:={\left(\Pi +H^{T}H\right)}^{-1}H^{T},$$

where $\Pi +H^{T}H$ resulted positive definite. For more details, see [35].

## 6. Aberration correction results

In the following section we report the results of correcting specimen-induced aberrations in three different samples using the generalised PN algorithm outlined in Section 4. We chose P=7 for the first two dimmer samples and P=5 for the HeLa cell sample. An in-depth discussion about the trade-offs concerning the selection of P is found elsewhere [24]. We set J=5 and neglected the first detail coefficient in Eq. (1), as it was mostly sensitive to Poisson noise. The wavelet scales are computed as $S_{j}=\delta_{P}\cdot 2^{j}$, where j is the index in Eq. (1) and $\delta_{P}$ is the pixel size. For $\delta_{P}=50$ nm, the selection above corresponds to the scales of 0.1, 0.2, 0.4, and 0.8 μm. First, correction of the spherical mode (Noll index 11) was performed, by acquiring images within the longitudinal xz plane. After that, we corrected Zernike modes with Noll indices between 5 and 10 inclusive, by acquiring images in the lateral xy plane. A parabola was used as the unimodal fitting function, and the fits were obtained by applying a trust-region optimisation algorithm [36]. The bias aberrations were set to 123 nm, which corresponds to 1 radian at the depletion wavelength of 775 nm. After identifying a suitable region of interest (ROI), the generalised PN algorithm was applied once in confocal mode and a second time in STED mode, using the first application as an initial condition. Except from the initial correction of static aberrations after mounting the sample, all reported AO was performed using the DM only. All aberration correction results reported within this paper were obtained using the same generalised PN algorithm and the parameters outlined above. The cumulative number of images acquired to correct both in confocal and in STED mode is therefore 2PN. Since the microscope features a resonant scanner with a fixed period, the pixel dwell time is not constant throughout a line scan and is also dependent on the number of pixels in the line. The dwell time for the experiments below was typically between 0.07 and 0.35 μs. No frame accumulation was used, whereas line accumulation was typically set between 20 and 200, depending on the brightness of the sample.

Before mounting any sample we first verified the correct axial and lateral alignment of the depletion and excitation foci by imaging gold beads in reflectance mode, i.e., temporarily enabling the branch that includes MMF3 in Fig. 4. Misalignment of the foci was manually corrected by applying tip, tilt, and defocus to the holograms displayed on the SLM. Subsequently, we applied sensorless AO to remove any static aberration present in the system. As a further verification, we performed these same steps while imaging a fluorescent beads sample.

**Fig. 5.**
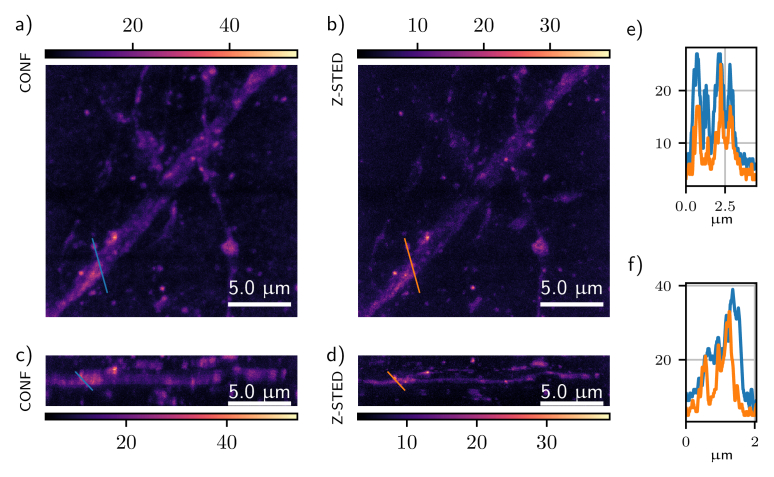
Maximum projections of CA1 pyramidal neurons filled with Alexa 594 in an organotypic hippocampal slice imaged at a depth of approximately 8 μm. The projections are computed by summing the raw photon counts of a 20×20×4 μ$m^{3}$ volume (400×400×80 pixels). (a) and (b) xy projections acquired in confocal and Z-STED mode, respectively; (c) and (d) xz projections; (e) profiles of the lines marked in the xy projections, computed using cubic interpolation; (f) profiles of the lines marked in the xz projections. Static aberrations have been corrected.

The first sample shows CA1 pyramidal neurons filled with Alexa 594 in an organotypic hippocampal slice, which we imaged using the orange channel (MMF2 in Fig. 4). For this sample we used 594 nm excitation with an average power of 1–6.6 μW measured at the BA of the microscope objective. After mounting the sample, we imaged the shallowest neurons at a depth of approximately 8 μm from the coverslip, and applied sensorless AO in confocal mode to ensure that the microscope was still operating close to aberration-free. Note that in this step we removed both any static aberration due to the mounting of the sample and any specimen-induced aberration for the current depth and ROI. Figure 5 shows the maximum projections from a 20×20×4 μ$m^{3}$ volume imaged both in confocal mode (on the left) and Z-STED mode (on the right). In this latter case, only one of the two holograms of the SLM was enabled to display the Z-STED phase mask. Throughout the whole paper, we used average STED powers of 30–170 mW measured at the BA of the objective.

**Fig. 6.**
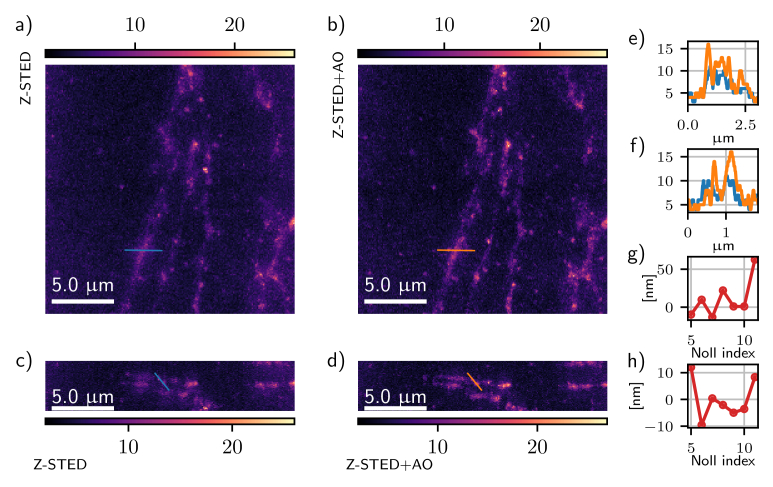
Maximum projections of CA1 pyramidal neurons imaged in Z-STED mode before and after AO at a depth of approximately 14 μm. The projections are obtained from the raw photon counts of a 20×20×4 μ$m^{3}$ volume (200×200×80 pixels). (a) and (b) xy projections before and after AO, respectively; (c) and (d) xz projections; (e) profiles of the lines marked in the xy projections; (f) profiles of the lines marked in the xz projections; (g) Zernike aberrations corrected in confocal mode. The coefficients are enumerated and normalised according to Noll [23], and given in units of nm; (h) Zernike aberrations corrected in Z-STED mode, obtained using the preceding confocal correction as an initial condition.

We subsequently moved deeper into the sample and imaged a 20×20×4 μ$m^{3}$ ROI at approximately 14 μm depth. We first applied AO in confocal mode, then switched into Z-STED mode and applied a second round of AO using the confocal correction as an initial condition. The results are reported in Fig. 6, which shows the xy and xz projections before (on the left) and after (on the right) applying the two rounds of AO. The corrected aberrations are reported in Fig. 6(g) (confocal mode) and Fig. 6(h) (Z-STED mode) in nanometers. From the figure it can be concluded that the algorithm is able to correct large amounts of aberration in confocal mode, whereas a finer correction is obtained after switching into Z-STED mode. At the same time, successful aberration correction is achieved using the same metric and settings for both confocal and Z-STED mode. This is in contrast with previously reported results [17,19], which required ad-hoc configurations for confocal and STED mode as well as different metrics.

**Fig. 7.**
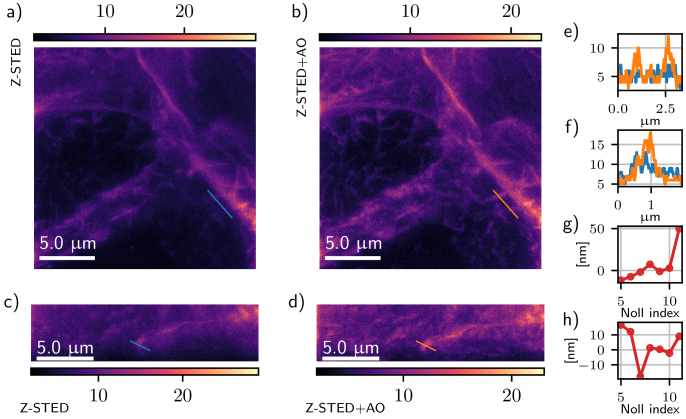
Maximum projections of actin labelled with silicon rhodamine in COS-7 cells imaged in Z-STED mode before and after AO at a depth of approximately 6 μm. The projections are obtained from the raw photon counts of a 20×20×5 μ$m^{3}$ volume (500×500×120 pixels). (a) and (b) xy projections before and after AO, respectively; (c) and (d) xz projections; (e) profiles of the lines marked in the xy projections; (f) profiles of the lines marked in the xz projections; (g) Zernike aberrations corrected in confocal mode; (h) Zernike aberrations corrected in Z-STED mode, obtained using the preceding confocal correction as an initial condition.

The steps outlined above were repeated for the second sample, which consists of actin filaments in COS-7 cells labelled with silicon rhodamine. We imaged this sample with the red channel (MMF1 in Fig. 4) using 657 nm excitation with an average power of 3.4 μW at the BA. The results are reported in Fig. 7, where we imaged a 20×20×5 μ$m^{3}$ volume at a depth of approximately 6 μm in Z-STED mode. Noticeable improvement can be seen in the brightness and contrast of the projections after AO has been applied. In addition, the aberration correction reveals some structures that are not otherwise discernable, as can be seen by examining the line profiles marked in the projections.

**Fig. 8.**
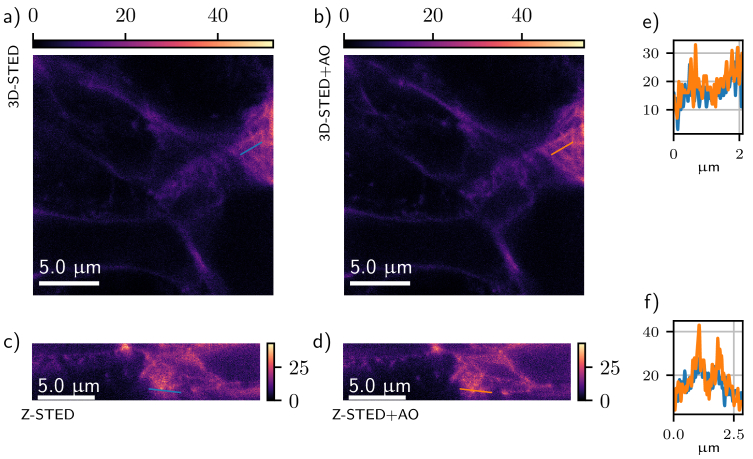
Comparison before and after the correction reported in Fig. 7. (a) 20×20 μ$m^{2}$ xy cross section (500×500 pixels) imaged in 3D STED mode before AO; (b) same as in (a) after AO; (c) 20×5 μ$m^{2}$ xz cross section (500×120 pixels) imaged in Z-STED mode before AO; (d) same as in (c) after AO. (e) profiles of the lines marked in (a) and (b); (f) profiles of the lines marked in (c) and (d).

To further assess the quality of the aberration correction reported in Fig. 7, we switched the microscope into 3D STED mode, i.e, we enabled both holograms on the SLM, one displaying the 2D enhancement phase mask and the other the Z one. We then acquired an xy cross section from an adjacent ROI both without and with the aberration correction obtained for Fig. 7. The results are reported on the top row of Fig. 8, which shows a 20×20 μ$m^{2}$ xy cross-section before and after AO. Notable improvement in the contrast can be seen even though the aberration correction was previously obtained using the Z-STED modality. Improvement along the axial direction can also be seen examining Figs. 8(c) and (d), which show xz cross-sections before and after the same AO.

**Fig. 9.**
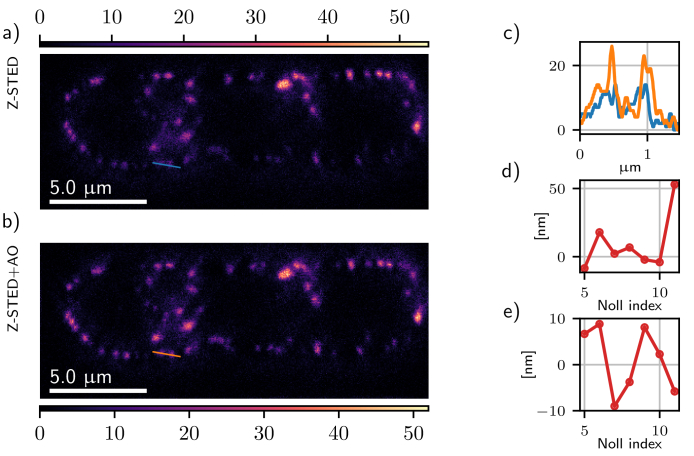
HeLa cell nucleus in mid S-phase with replication sites labelled by a short EdU pulse and detected with Alexa Fluor 594. The imaging mode is Z-STED. (a) 20×8 μ$m^{2}$ xz cross section (400×160 pixels) before AO; (b) same as (a) after AO; (c) profiles marked in the cross sections; (d) Zernike aberrations corrected in confocal mode; (e) Zernike aberrations corrected in Z-STED mode, obtained using the preceding confocal correction as an initial condition.

The final sample consisted of a HeLa cell nucleus in mid S-phase with replication sites labelled by a short EdU pulse and detected with Alexa Fluor 594. We imaged this sample with the orange channel using 594 nm excitation with an average power of 4–6 μW at the BA. After following the steps outlined for the first sample, we imaged a 20×8 μ$m^{2}$ xz cross section before and after applying AO. The results are reported in Fig. 9. The improvement due to the AO is visible by examining the deeper region at the bottom marked with the line profiles. Here the labelled structure is only clearly discernable after AO.

## 7. Conclusions

In this paper we introduce a novel wavefront sensorless aberration correction algorithm that is based on wavelet analysis. The salient feature of this algorithm is its capability to perform spatially localised frequency analysis of images, which stems directly from the use of wavelets. This is in contrast with previously proposed methods that are entirely based on Fourier analysis [10,19,25]. As a result, we are able to establish a vector-valued generalisation of the image quality metric, which allows to track spatially localised frequency variations as a function of the residual aberration in a microscope. To achieve this, we have proposed a straightforward generalisation of the PN algorithm [24] that can be deployed in our context. Finally, we have validated our approach via aberration correction experiments on a custom-built stimulated emission depletion (STED) microscope using three different biological specimens. Our results show that our technique is successful in removing aberration when the microscope is operating both in confocal mode and in STED mode. This overcomes the necessity to use different approaches for these two modalities [8,10,19]. In addition, it obviates the need for empirical tuning of the parameters of the metric when different samples are imaged [19]. Our results bear also significance in the broader context of adaptive optics for microscopy, as we have shown that a single algorithm is able to achieve aberration correction in two distinct microscopy techniques, namely confocal and STED microscopy. This is a crucial step in developing a unified approach that can facilitate widespread adoption of this technology. We expect that our method would be ideally suited for further development with machine learning techniques [37], where aberration correction would be achieved by more sophisticated tracking of the variations of the wavelets coefficients at different scales and, additionally, combined with denoising.

## Data Availability

The research materials supporting this publication can be accessed at https://doi.org/10.5287/bodleian:7RzzNpw4P.
